# Computational Characterization of Modes of Transcriptional Regulation of Nuclear Receptor Genes

**DOI:** 10.1371/journal.pone.0088880

**Published:** 2014-02-13

**Authors:** Yogita Sharma, Chandra Sekhar Reddy Chilamakuri, Marit Bakke, Boris Lenhard

**Affiliations:** 1 Department of Biomedicine, University of Bergen, Bergen, Norway; 2 Department of Clinical Medicine, University of Bergen, Bergen, Norway; 3 Institute of Clinical Sciences, Faculty of Medicine, Imperial College London, and MRC Clinical Sciences Centre, London, United Kingdom; 4 Department of Informatics, University of Bergen, Bergen, Norway; Philipps University, Germany

## Abstract

**Background:**

Nuclear receptors are a large structural class of transcription factors that act with their co-regulators and repressors to maintain a variety of biological and physiological processes such as metabolism, development and reproduction. They are activated through the binding of small ligands, which can be replaced by drug molecules, making nuclear receptors promising drug targets. Transcriptional regulation of the genes that encode them is central to gaining a deeper understanding of the diversity of their biochemical and biophysical roles and their role in disease and therapy. Even though they share evolutionary history, nuclear receptor genes have fundamentally different expression patterns, ranging from ubiquitously expressed to tissue-specific and spatiotemporally complex. However, current understanding of regulation in nuclear receptor gene family is still nascent.

**Methodology/Principal Findings:**

In this study, we investigate the relationship between long-range regulation of nuclear receptor family and their known functionality. Towards this goal, we identify the nuclear receptor genes that are potential targets based on counts of highly conserved non-coding elements. We validate our results using publicly available expression (RNA-seq) and histone modification (ChIP-seq) data from the ENCODE project. We find that nuclear receptor genes involved in developmental roles show strong evidence of long-range mechanism of transcription regulation with distinct *cis*-regulatory content they feature clusters of highly conserved non-coding elements distributed in regions spanning several Megabases, long and multiple CpG islands, bivalent promoter marks and statistically significant higher enrichment of enhancer mark around their gene loci. On the other hand nuclear receptor genes that are involved in tissue-specific roles lack these features, having simple transcriptional controls and a greater variety of mechanisms for producing paralogs. We further examine the combinatorial patterns of histone maps associated with dynamic functional elements in order to explore the regulatory landscape of the gene family. The results show that our proposed classification capturing long-range regulation is strongly indicative of the functional roles of the nuclear receptors compared to existing classifications.

**Conclusions/Significanc:**

We present a new classification for nuclear receptor gene family capturing whether a nuclear receptor is a possible target of long-range regulation or not. We compare our classification to existing structural (mechanism of action) and homology-based classifications. Our results show that understanding long-range regulation of nuclear receptors can provide key insight into their functional roles as well as evolutionary history; and this strongly merits further study.

## Introduction

Nuclear receptors comprise one of the largest groups of transcription factors that regulate the activity of complex gene networks [1], [2], [3]. These genes work in concert with co-activators and co-repressors to regulate a wide variety of biological processes such as embryonic development, organogenesis and metabolic homeostasis [4], [5]. Improper functioning of nuclear receptors has been implicated in various developmental and physiological disorders [6], and nuclear receptors are known to be promising drug targets [7], [8].

Nuclear receptors are broadly classified either based on their sequence similarity [9] or depending on their ligands [10]. Based on sequence homology, nuclear receptors have been categorized into 7 subclasses [9]. Alternatively, nuclear receptors are classified as nuclear hormone receptors (NHR) or nuclear orphan receptors (NOR) based on their mechanism of action. Nuclear hormone receptors are activated via ligand binding, but ligand binding by nuclear orphan receptors has not been demonstrated [11] and their mechanism of action is poorly understood. Some studies have reported that they are activated by post-translational modification or direct transcriptional activation [12], [13]. Furthermore, some nuclear receptors have been categorized into tissue-specific and developmental regulatory based on their known functional roles [14], [15], [16].

Early research explored the structural properties of nuclear receptors [17], while recent work has focused on understanding how individual nuclear receptors control the transcription of their target genes [18], [19], [20], [21]. However, how nuclear receptors are themselves regulated (rather than how they regulate their target genes) is not well understood [22], [23]. This leads to the following question: Does regulation of nuclear receptor genes exhibit characteristic behavior in terms of their sequence similarity, mechanism of action or functional roles? Understanding regulation of nuclear receptors promises fresh insight into the functional roles of these genes, and possibly, accounting for at least a subset of disease-associated variation found in their vicinity.

In this paper, we hypothesize that the diversity of the biological and biochemical roles of nuclear receptors is reflected in fundamental differences in their transcriptional regulation e.g. whether the nuclear receptor in question is a target of long-range regulation or not. Like many other genes specific for one tissue, tissue-specific ligand-modulated nuclear receptors are expected to have relatively simple transcriptional control: they will be turned on in their target tissue only, and consequently, may not be targets of long-range regulation. On the other hand, nuclear receptors involved in developmental processes should exhibit properties that have been established for developmentally regulated genes [24]. These properties include long-range control of gene regulation by highly conserved non-coding elements and multiple long CpG islands. The highly conserved non-coding elements form clusters in a large region around their target gene loci and can function as enhancers [25].

It has been proposed that nuclear receptors first appeared as a single gene that has duplicated and diversified into current seven subfamilies during evolution [26]. We hypothesize that in many cases, it is the ancestral and not the currently extant gene loci that have been recruited into the developmental or the tissue-specific roles. Those functions were then passed to their duplicate offspring loci, which then sub-functionalized or acquired entirely new functions with different mode of regulation.

**Figure 1:**
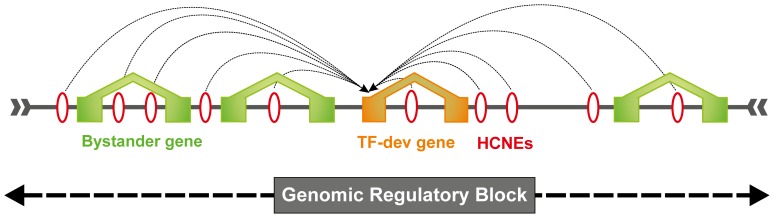
The GRB Model. GRB has developmental and/or transcription factor gene (target gene, orange) spanned by a cluster of highly conserved non-coding elements (red ovals), which regulates the target gene expression by acting as enhancers/insulators and other un-related neighboring genes (bystander genes, green).

In this study of the nuclear receptor gene family, our aim was to establish whether or not they possess properties that would classify them as targets of long-range developmental regulation, and analyzed the relationship between their *cis*-regulatory content and their known functions. To facilitate this work we used an established genomic regulatory block (GRB) model [27], [28]. A GRB is a locus on a chromosome that carries all the regulatory input required for the expression of a ‘target’ gene. This block comprises a target gene, its enhancers including highly conserved non-coding elements (HCNEs) and often bystander genes. Target genes receive regulatory input from HCNEs, which can be present either in inter- or intra-genic regions (Figure 1). Bystander genes contain HCNEs in their introns or beyond, but do not respond to their regulatory input; these HCNEs also control the target gene resulting in conservation of synteny between the two genes as a by-product of maintaining the organization of GRBs, which needs to be conserved for the normal functioning of the target gene [29], [30].

Our first aim was to establish which genes among the nuclear receptors are potential GRB target genes. We then investigated the impact of the *cis*-regulatory content of each gene in order to gain a deeper understanding of its transcriptional regulation. Using publicly available datasets from the ENCODE project [31], we considered histone modifications known to be associated with promoters, enhancers, transcriptional repression and transcription elongation. Finally, to understand the complete regulatory landscape of nuclear receptors, we used chromatin states map data obtained by ChromHMM segmentation on ENCODE cell lines [32], consisting of the genome-wide combinatorial patterns of various histone marks, which are known to be associated with distinct biological functions [33]. We studied the enrichment pattern of all the defined chromatin states in nuclear receptors in the H1 human embryonic stem cell line (H1hesc). We define a new classification of nuclear receptor genes on the basis of their transcriptional regulation, and show that nuclear receptors naturally fall into two clusters: one comprising GRB target genes, i.e. developmental regulators that maintain a complex pattern of expression; and one comprising non-target genes that require simpler transcriptional control. The evolutionary history of nuclear receptor genes shows the differential use of whole-genome versus gene duplications between the two groups. This study will aid in better understanding of the regulatory mechanism of nuclear receptor genes and their functional diversity.

## Results

### Classification of Nuclear Receptors with Respect to GRB Model

Our first aim was to determine which nuclear receptor genes possess the properties of GRB target genes. To facilitate this, we analyzed the HCNE regions around each nuclear receptor gene locus across five vertebrate genomes. Since it has been shown that most HCNEs act as long-range enhancers of their target genes [34], we analyzed HCNEs in 1 Mb or 2 Mb span upstream and downstream of gene loci, using custom levels of conservation for different species. To maximize the information from the set of elements for each of the selected vertebrate species, the conservation threshold for different species was chosen between 70 to 100 percent, depending on the evolutionary distance from human (see Table S1 for details). We calculated HCNE counts around 2 Mb region of each nuclear receptor gene loci.

**Figure 2:**
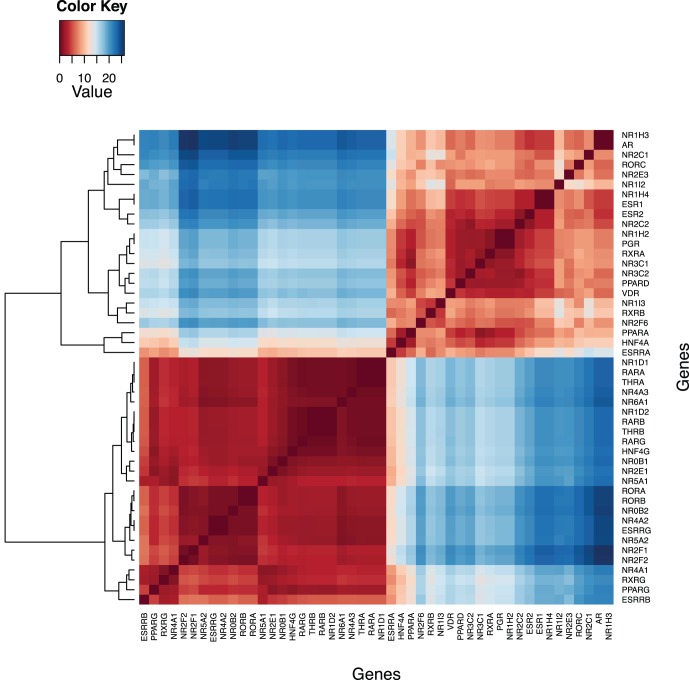
The dissimilarity matrix of HCNE content among nuclear receptors and its clustering. Nuclear receptor genes broadly divided in to two clusters on the basis of higher and lower enrichment of HCNEs around 2(shown below) consists of 25 genes having higher enrichment of HCNE, while cluster 2 consists of the remaining 23 genes.

Detection of HCNE regions was the first step towards identifying which genes in the nuclear receptor family have the features of GRB target genes. We computed dissimilarity matrix of HCNEs between human and five selected vertebrate genomes and performed the hierarchical clustering (see Methods section on “HCNE and CpG islands detection”). We found that whole gene family can be broadly divided into two main clusters containing 25 and 23 genes respectively (Figure 2).

Table 1 shows the list of genes in the two clusters as well as their functional and structural classification. The genes in cluster 1 have a higher span of HCNEs around their gene loci, whereas cluster 2 genes have few or no HCNEs (Table 1). Interestingly, the first cluster comprises of many genes that are known targets of long-range gene regulation (e.g. *NR2F2*, *PPARG* [24]). Thus, cluster 1 corresponding to high HCNE counts in the GRB model is indicative of possible targets of long-range gene regulation. In the sequel, we explore this hypothesis further by considering other promoters and *cis*-regulatory elements.

**Table 1:** The list of genes in clusters obtained using HCNE based analysis in the GRB model.

Gene Name	Cluster ID	Homology-based subfamily	Mechanism of action
NR1D1	1	I	NHR
RARA	1	I	NHR
THRA	1	I	NHR
NR4A3	1	IV	NOR
NR6A1	1	VI	NOR
NR1D2	1	I	NHR
RARB	1	I	NHR
THRB	1	I	NHR
RARG	1	I	NHR
HNF4G	1	II	NHR
NR0B1	1	0	NOR
NR2E1	1	II	NOR
NR5A1	1	V	NHR
RORA	1	I	NHR
RORB	1	I	NHR
NR0B2	1	0	NOR
NR4A2	1	IV	NOR
ESRRG	1	III	NOR
NR5A2	1	V	NHR
NR2F1	1	II	NOR
NR2F2	1	II	NOR
NR4A1	1	IV	NOR
RXRG	1	II	NHR
PPARG	1	I	NHR
ESRRB	1	III	NOR
NR1H3	2	I	NHR
AR	2	III	NHR
NR2C1	2	II	NOR
RORC	2	I	NHR
NR2E3	2	II	NOR
NR1I2	2	I	NHR
NR1H4	2	I	NHR
ESR1	2	III	NHR
ESR2	2	III	NHR
NR2C2	2	II	NOR
NR1H2	2	I	NHR
PGR	2	III	NHR
RXRA	2	II	NHR
NR3C1	2	III	NHR
NR3C2	2	III	NHR
PPARD	2	I	NHR
VDR	2	I	NHR
NR1I3	2	I	NHR
RXRB	2	II	NHR
NR2F6	2	II	NOR
PPARA	2	I	NHR
HNF4A	2	II	NHR
ESRRA	2	III	NOR

The homology-based classification is into seven categories: (I) Thyroid Hormone Receptor-like, (II) Retinoid X Receptor-like, (III) Estrogen Receptor-like, (IV) Nerve Growth Factor IB-like, (V) Steroidogenic Factor-like, (VI) Germ Cell Nuclear Factor-like, and (0) Miscellaneous. The functional classification is into nuclear hormone receptors (NHR) and nuclear orphan receptors (NOR).

We observe that the genes are dispersed among the two clusters irrespective of their homology-based classification (Table 1), indicating that following duplication events in evolutionary history, one of the genes acquired a different mode of regulation. However, we observe that most recent paralog pairs of genes (e.g. *NR2F2* and *NR2F1*; *NR5A2* and *NR5A1*) reside in the same cluster, with few exceptions (e.g. *PPARG* and *PPARA; NR2E1* and *NR2E3*). Indeed, close paralogs belonging to the first cluster can be traced back to one of the two rounds of whole-genome duplication that happened at the root of vertebrates. This is naturally indicative that the genes in the first cluster having high HCNE counts have possibly evolved through whole-genome duplication rather than tandem duplication. Due to the megabase span of their regulatory regions, it is practically impossible for GRB target genes to undergo tandem duplication without disrupting the array of associated regulatory elements.

**Figure 3:**
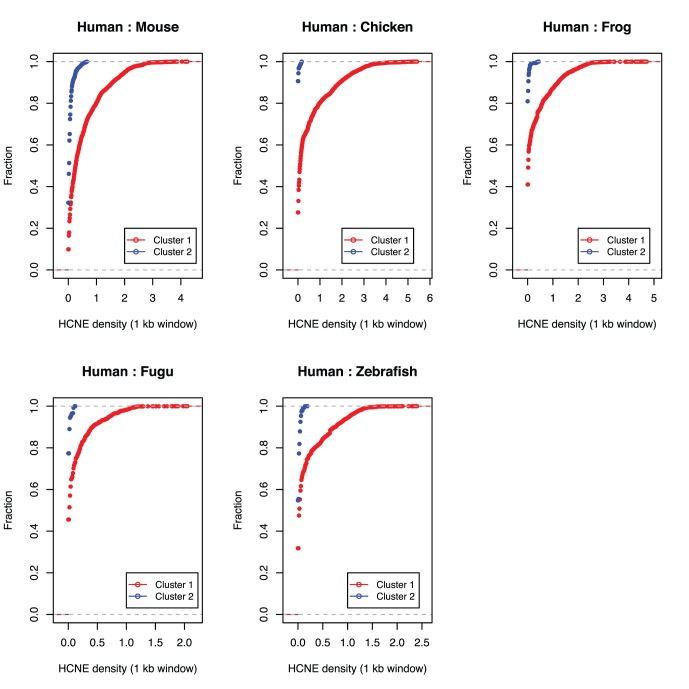
Cumulative distribution plots of HCNE content for human versus 5 vertebrate genomes in 2 Mb region from gene loci across different clusters. Cluster 1 (putative GRB target genes) is shown in red and cluster 2 (GRB non-target genes) is shown in blue. The x-axis shows HCNE distribution in 1 kb window and y-axis show the fraction of HCNE in selected window. This figure shows that Cluster 1 has higher fraction of HCNEs in comparison to cluster 2.

The above analysis is based on the genomes of five species. To understand the variation within species, we perform subsequent analysis by comparing HCNE counts among each species to human. We visualized HCNEs of each gene loci across 2 Mb region using 1 kb windows in the two clusters (Figure 3). We observe that the genes in cluster 1 (shown in red) have a higher number as well as a wider span of HCNEs around their gene loci in comparison to the genes in cluster 2 (shown in blue). Both the number and the maximum span of HCNEs decreased with increasing evolutionary distance from human, e.g. human-mouse compared to human -zebrafish. However, the number of HCNEs decreases with increasing evolutionary distance but still does not completely disappear in cluster 1 even at the highest investigated distance i.e. human-zebrafish.

It has been shown earlier that GRB target genes often have higher ratios between CpG island length and transcript length [25]. In contrast to most other genes, CpG islands in GRB target genes not only cover the promoter region but also extend into the body of the gene, in some cases, spanning the entire target gene. Therefore we checked the CpG islands around gene loci in cluster 1 and 2 and found that most of the genes in cluster 1 have longer CpG islands in comparison to cluster 2 (Wilcoxon test, p-value <0.0001), confirming that the high HCNE counts and multiple long CpG islands are correlated features of the genes present in cluster 1. Since we are analyzing the length of CpG islands among genes; we excluded the genes that do not overlap with any CpG island in both clusters. We also checked the CpG length of putative GRB target nuclear receptors (cluster 1) with randomly selected transcription factor genes, and with the set of all genes overlapping CpG islands. From the cumulative distribution plots (Figure S1), it is clear that GRB target nuclear receptors have longer CpG islands than the other sets.

### Extended Validation based on other Transcription Factors

To further validate the two classes, we compared the HCNE counts of the nuclear receptor gene family with other transcription factors. Specifically, we created a random dataset of 48 transcription factor genes and computed the HCNEs across the five vertebrate genomes (see Methods for details). We repeated previous experiment using the extended set of 96 genes (48 nuclear receptors and 48 randomly selected transcription factors) with the same distance and conservation threshold as before. We found that the extended set was divided into two major clusters (Figure S2 and Table S2). The first cluster comprised of 31 genes in total, out of which 25 are nuclear receptors and 6 are other transcription factors (Cluster A in Table S2). The second cluster has 65 genes, 23 of which are nuclear receptors and 42 are other transcription factors (Cluster B). The resulting clustering agrees with previous results i.e. the genes that clustered together in previous HCNE analysis (cluster 1 in Table 1) are part of the same cluster here (cluster 1 in Table S2). Interestingly, we also found other transcription factors (*PAX2, SOX2, MEIS2*) in this cluster that are known targets of long-range gene regulation [36], [37], [38]. This shows that the previous clustering is robust and functionally significant, and more generally, that this method can be used to study other developmental regulated genes as well.

### Identification of Target Nuclear Receptor in GRB Loci having Several Genes

In the previous analysis (Table 1), we found three cases of GRB loci with several target genes appearing in cluster 1, namely (*THRB, RARB, NR1D2),* (*THRA, RARA, NR1D1*), and (*NR6A1*, *NR5A1*) wherein the genes in each case share a common locus w.r.t. HCNEs within a ±2 Mb region. In such a scenario, it is not immediately clear which of the gene (or genes) is the target in the corresponding GRB locus. Investigating further, we found that in each of the cases above, the genes are present in synteny in human and mouse (see Figure S3) – lending further credence to the idea that these genes were part of whole-genome duplication.

However, the problem of identifying target genes in a GRB locus remains. While proximity of each gene to HCNE peaks offers some indication, it is not sufficient. In the sequel, we report experiments based on expression and histone-modification data in the H1hesc embryonic stem cell line. The results (which are described in more detail later in the manuscript) address the afore-mentioned problem based on presence of bivalent domain in the promoter region of the gene.

In the first case, *RARB* was located most closely to the peaks of highest HCNEs and also it has bivalent promoter (though very weak) in H1hesc cell line. On the other hand, the genes *NR1D2* and *THRB* have neither a proximal HCNEs peak (in comparison to other common gene in GRB locus) nor a bivalent promoter. Therefore, we annotate *RARB* to be the putative target of this GRB locus. In the second case, all the three genes (*THRA, RARA, NR1D1*) shares the same proximity of HCNEs around each other but only two (*RARA* and *NR1D1*) have bivalent promoters; therefore we annotated these two as targets of the same GRB locus. (Both of these follow same expression pattern in rest of the cell lines). In the third case, both *NR6A1* and *NR5A1* exhibit similar proximity of HCNEs but neither have a bivalent domain. In this case, the *NR6A1* gene is already highly expressed in H1hesc cell line in comparison to other expressed genes, while gene *NR5A1* is completely shut down. Therefore we annotated both of these genes as putative targets of the GRB.

### Distinct Expression Profiles of Cluster 1 and Cluster 2 Genes

To investigate the expression properties of cluster 1 and cluster 2 genes, we used read per kilobase per million (RPKM) values for each gene from RNA-seq data across 5 ENCODE cell lines (Table S3). Based on this, we categorized each gene set on the basis of expression significantly above the background (RPKM = 0.3) in respective cell lines, following approach in [39]. The total number of genes expressed across different cell lines was highest in the H1hesc and HepG2 cells. For each cell line, we considered four sets of genes obtained on the basis of their expression significantly above and below the background across both the clusters.

We observe that most genes belonging to cluster 1 are expressed in H1hesc (Table S3) and had relatively lower RPKM with few exceptions. On the other hand, the genes in cluster 2 had either expression in one cell line (e.g. *HNF4A,* and *NR1H4* were specific for HepG2 cell line) or they had very high expression values across all the cell lines (e.g. *NR2C2* and *NR2C1*). This shows that the clustering likely separates developmentally regulated genes from all other genes (ubiquitous and tissue specific) in line with the ability of their promoters to respond to long-range regulation [40].

### H3K4me3 and H3K36me3 Enrichment Confirms Expression-based Analysis

To check the expression status of genes, it was crucial to check if the selected RPKM threshold of 0.3 actually correlates with the histone marks of expressed genes. To confirm this, in both clusters we studied the enrichment profiles of histone modification that relates to active promoter (H3K4me3) in respective cell lines (see section on “ChIP-seq data” in Methods for details). We selected ±10 kb region around transcription start sites for the analysis and plotted the coverage. We found the enrichment of active promoter mark peaks in promoter region of genes expressed significantly above the background across both the cluster 1 and cluster 2 gene sets. No enrichment was observed when the genes are in low expression state (Figures S4 and S5).

We also analyzed the enrichment of transcription elongation mark (H3K36me3) across genes in both the clusters (see section on “ChIP-seq data” in Methods for details). To be able to handle the difference in gene coordinates, we used ±20 kb genomic ranges around the midpoint of each gene where the midpoint is chosen to be the mean of the gene start and end coordinates. The enrichment of transcription elongation mark was observed across the gene body of only those genes that express significantly above the background in both the clusters in their respective cell lines; there was no enrichment when genes are low expressed. Both of these analyses confirm the main objective and showed the accuracy of expression state of gene sets created on the basis of selected threshold value.

### Loci of Cluster 1 Genes have Significantly Higher Enrichment of H3K4me1

We are mainly interested in exploring the differences in regulatory content of genes with respect to their functions; those involved in developmental regulation must be under long-range control. Therefore, we analyzed the enrichment profiles of histone modification (H3K4me1) in H1hesc stem cell line (see section “ChIP-seq data” in Methods), a modification associated with active and poised enhancers. For H3k4me1 analysis across the different clusters, we did not consider the expression state of genes in respective cell lines, as its already shown in various studies that this mark is related to active and poised enhancer, and is not predictive of current transcription state.

We plotted the average coverage plots ±50 kb around transcription start site (TSS) for both of the clusters. We chose ±50 kb as a compromise value between establishing the existence of long-range regulation and avoidance of inclusion of regulatory elements of neighboring genes. We found that cluster 1 has higher enrichment of enhancer marks in comparison to cluster 2.

To check whether the observed difference is statistically significant, we created background distribution of H3K4me1 number of reads as well as specific datasets of CpG-overlapping and non-CpG promoters (see Methods for details). We study enhancer mark for each dataset with respect to this background distribution across different genomic ranges (see Methods for details).

**Figure 4:**
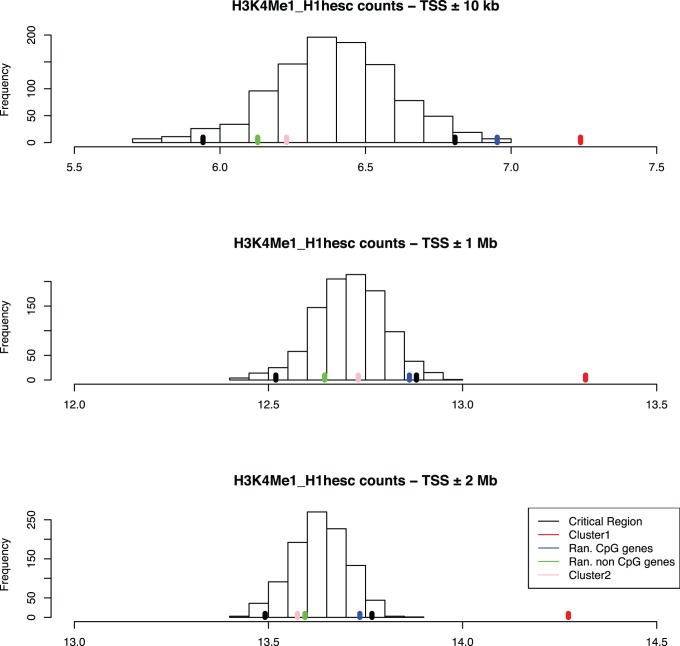
Statistical significance test for H3K4me1 around different genomic distributions. A) H3K4me1 distribution in different clusters across ±10 kb TSS against the random background distribution. B) H3K4me1 distribution in different clusters across ±1 Mb TSS with respect to random background distribution. C) H3k4me1 distribution in different clusters across ±2 Mb TSS with respect to random background distribution. This figure shows that cluster 1 (shown by red bar) has significantly higher distribution of H3K4me1 in comparison to random selected background region (marked by black bars), CpG and non-CpG region (shown by blue and green bar respectively) and cluster 2 genes (shown by pink bar).

Figure 4 shows the distribution of reads for each of the selected genomic ranges (respectively, ±10 kb, ±1 Mb and ±2 Mb). We define the critical region for each of the chosen widths by considering log2 value computed from the 0.95-quantile of the corresponding background distribution. Finally we check the occurrence of each dataset with respect to this critical region by considering log2 value of the average number of reads in each of the four original datasets, namely, nuclear receptors in clusters 1 and 2, as well as background set with and without CpG-islands.

We find that for each genomic range under consideration (respectively, ±10 kb, ±1 Mb and ±2 Mb), cluster 1 consistently falls well outside the critical region of the corresponding background distribution (Figure 4). We also observe that the set of CpG genes falls outside of critical region when we consider a region of ±10 kb around TSS. This concurs with the fact that in general CpG genes tend to have higher enrichment of H3k4me1 around their promoter region in comparison to non-CpG genes. However, when we consider ±1 Mb and ±2 Mb genomic regions; three of the four sets of gene, namely, cluster 2, the set of CpG genes, and the set of non-CpG genes, fall within the critical region of the background distribution. This analysis clearly shows that cluster 1 genes have statistically significant higher enrichment of enhancer mark around ±1 Mb and ±2 Mb of their transcription start site, indicating that they follow long-range mechanism of gene regulation, unlike the genes of cluster 2. To exclude the possibility of bias, we have also repeated the experiment by using genes on chromosome 5 for the background distribution. We found that genes in cluster 1 still have significantly higher enrichment of H3K4me1 across the different genomic ranges (Figure S8).

### Cluster 1 Genes have Bivalent Promoters in H1hesc Stem Cell Line

It is known that genes involved in developmental regulation have bivalent promoters in stem cells [41], which means they have both active (H3K4me3) and repressive (H3K27me3) histone mark enrichment on the same locus. The presence of bivalent promoter mark enables these genes to turn on and off rapidly across different time points of development [41]. The bivalent state indicates a repressed state poised for activation. On activation, H3K27me3 is removed and only H3K4me3 remains. We were interested to test this observation across genes of both clusters in human embryonic stem cell line (H1hesc). We found that repression mark was completely absent in cluster 2 irrespective of their expression state in embryonic stem cell line, confirming that this cluster consists of a mixture of ubiquitously expressed genes and genes specifically expressed in later stages of differentiation.

The genes in cluster 1 consistently show evidence of involvement in developmental processes. We observed very high enrichment of repression mark around promoter region across genes in cluster 1 specifically when they are not expressed (Figure S6), showing that they have the type of promoter required to facilitate their complex pattern of expression.

**Figure 5:**
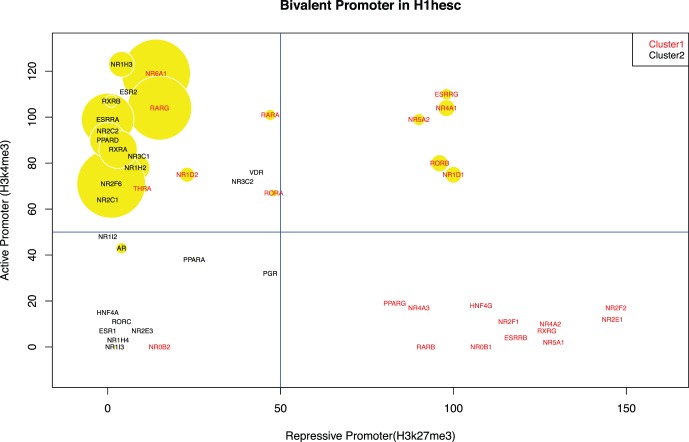
The bubble plots for bivalent promoter mark for each gene in human embryonic stem cell line. The x-axis shows read counts for repression (H3K27me3) mark around ±10 KB TSS. The y-axis shows read counts for active promoter (H3K4me3) mark around ±10 KB TSS. The size of the bubble (yellow) shows RPKM value for respective gene. The left section of the plot comprises all of the genes (black) in cluster 2 (except few cases where cluster 1 gene have very high expression). This shows that cluster 2 genes does not have any enrichment of repression mark around their TSS irrespective of their expression. The top and bottom right sections consist of genes from cluster 1 (red). This shows that when genes in cluster 1 are not expressed they have higher read counts for repression mark while still some of the genes retain repression mark even when they are expressed.

Figure 5 shows the correlation of the two promoter marks across both clusters, we plotted bubble plots for each gene showing H3K27me3 and H3K4me3 marks for each gene at x-axis and y-axis respectively, and the expression level (derived from RNA-seq RPKM values, see Methods for details) represented by the size of the bubble. The genes in cluster 2 (marked in black) do not have read counts for H3K27me3 repression mark even when they are not expressed, while on other hand genes in cluster 1 (marked in red) have very high read counts for repression mark when they are not expressed (appearing in bottom-right quadrant). This is consistent with our hypothesis that genes in cluster 2 do not have long-range regulation, and consequently, do not need a repressive promoter mark. On the other hand, we posit that genes in cluster 1 as targets of long-range regulation; and show high repressive mark pausing transcription and resulting in low expression (bottom-right quadrant in Figure 5).

We further notice a handful of genes in cluster 1 (*ESRRA, NR6A1, RARG, RORA, RARA*) do not have repression mark (appearing in top-left quadrant), while having high expression values (large bubbles in the plot). These genes are likely turned on early enough to be active in H1 hESC cells, but their expression pattern across other cell lines and H3K4me1 mark content at their loci still confirm that they are under developmental regulation.

The most interesting observation we make is that few genes in cluster 1 (*NR4A1, NR5A2, NR1D1, RORB* and *ESRRG*) still retain repression read counts even when they are actively transcribed (shown in top-right quadrant of Figure 5). We believe these genes represent the transition either from expressed and no repressive mark (top-left quadrant) to low expressed and high repressive mark (bottom-right quadrant), or vice versa. We further investigated how exactly the promoter region looked in these five cases (Figure S7). A closer look at promoter region reveals that in case of *NR1D1* and *RORB,* it seems like the promoter itself is not covered by the repression mark, which starts slightly downstream and extends into the first intron (Figure S7). The functional significance of this arrangement is unknown, but may represent a configuration conductive to rapid repression. The remaining three genes, namely *NR4A1*, *NR5A2* and *ESRRG*, also retain repression mark but are possibly transcribed from an alternative promoter. This merits further study possibly using time-series experiments in order to capture the dynamic activation and repression during development.

### GRB-based Clustering is Recovered from Chromatin State Map Analysis

To have better understanding of regulatory regions of nuclear receptors, we analyzed the chromatin state maps data for each gene in H1hesc cell line. This data represents the genome-wide mapping of different combinatorial patterns of histone marks, each of which is associated with specific biological function. The chromatin state map from [33] consists of 15 states, corresponding to the different functional elements of genome. To distinguish between active and repressed state of a gene, we also included the expression data in this analysis. For each nuclear receptor gene, we studied the correlation of different states with its expression.

**Figure 6:**
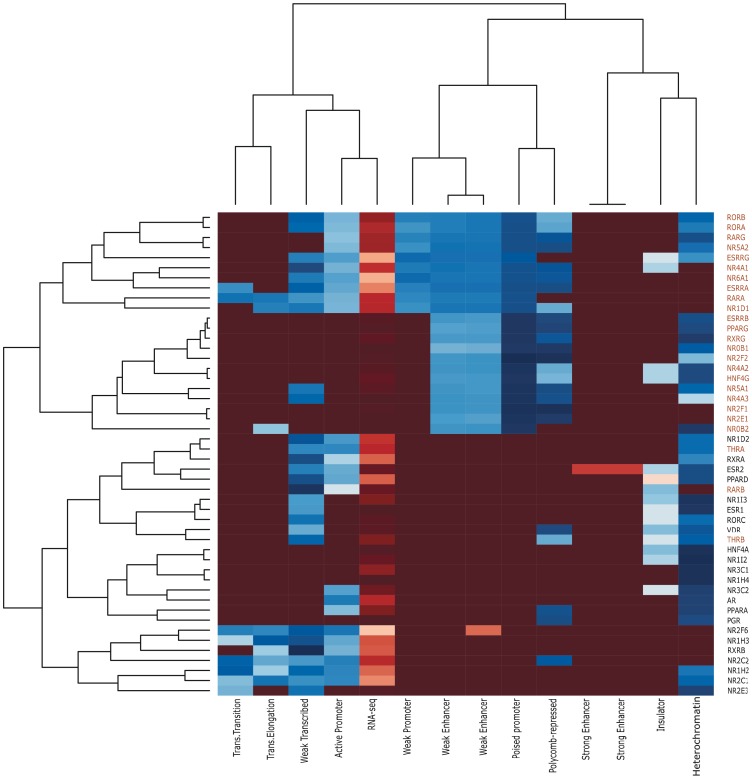
HMM state map analysis recovers the two clusters of nuclear receptor genes obtained using HCNE-based analysis. The columns of the heatmap show 13 different chromatin states alongwith RNA-seq data. The rows correspond to each nuclear receptor gene (Cluster 1 shown in red, Cluster 2 shown in black). The column and row side dendrogram represents the clusters of nuclear receptor genes on the basis of difference in their *cis*-regulatory functional elements and expression state.

Like in the case of previous analyses, we found that nuclear receptor genes separated into two major clusters on the basis of different enrichment of various chromatin states (Figure 6). The obtained clusters were based on the two main criteria: the expression status of the gene, and the difference in *cis*-regulatory functional elements. The column dendrogram shows that state correspond to active promoter correlates well with the expression (RNA-seq) data, which means that when genes are expressed significantly above the background they have higher number of counts for active promoter state and vice versa. The states that correspond to transcribed regions also correlate with the active promoter state, which confirms the presence of active transcription. The states that correspond to poised promoter and Polycomb repression occur together and are in a different column. Similarly the states that correspond to poised and weak enhancer show high correlation to each other, and so do the states that represent heterochromatin and insulator region. This shows that the column dendrogram corresponds well with the active biological functions.

However, in the row dendrogram i.e. at the gene level, nuclear receptors have broadly separated into two clusters, and each cluster is sub-classified in further two clusters depending on the expression level of the genes. The genes have different combinatorial patterns of states with respect to their expression state across the same cluster. We note that the obtained clustering based on HMM state map is consistent with the previous clusters found based on HCNE analysis (Table 1), with three exceptions, namely *THRA, THRB* and *RARB*. This is because GRB-based clustering takes into account the fact that these genes are in close proximity to other target genes, while HMM state maps do not take spatial proximity into account.

The genes present in cluster 1 exhibit enrichment of poised promoter state except three genes (*NR6A1, ESRRA, RARG*), because of their very high expression in this cell line. The genes having expression significantly above the background present in cluster 1 show enrichment of state that corresponds to active promoter and transcribed region, as well as higher enrichment of states that relates to weak enhancers. In contrast, the genes that do not have expression significantly above the background in cluster 1 are highly enriched in poised promoter state along with strong Polycomb repression and complete loss of active transcription states and RNA-seq signal.

Cluster 2 can be further sub-divided into two subclusters on the basis of expression level, but the associated states are distinct from those in cluster 1. The main difference lies in the enrichment of poised promoter and poised enhancer states. The genes present in cluster 2 are not associated with poised promoter or enhancer-related marks regardless of their expression state. This novel result further confirms the differences in regulatory mechanisms between the genes belonging to two clusters, indicating that cluster 1 (representing genes that are possible targets of long-range regulation) are the only ones that rely on poised configuration for rapid activation of gene expression.

## Discussion

Diverse functional roles of nuclear receptors and their direct/indirect involvement in physiological and developmental disorders and their potential as drug targets call for a better understanding of this important gene family. Insight into regulation mechanisms governing the transcription of nuclear receptor genes is central to this task. Further, this can provide clues towards the evolutionary history of nuclear receptors in question, e.g. recent paralogs sharing same mechanism of regulation are likely to have evolved through whole-genome duplication rather than tandem duplication. More fundamentally, analyzing the regulation mechanism for nuclear receptors can help decipher their diverse functional roles, and possibly accounting for genome variants found in their vicinity.

In this study, we investigated the properties of *cis*-regulatory environment of nuclear receptors towards understanding the diversity in their biological roles. The mode of transcription regulation of nuclear receptors is crucial for deciphering their function, which is not sufficiently captured by existing classifications of nuclear receptors based on their sequence homology [9] or mechanism of action.

Towards this goal, we have studied the *cis-*regulatory environment of each member of the gene family. We used the GRB model, which consists of target gene surrounded by highly conserved non-coding elements (HCNEs) and bystander genes, to analyze the neighborhood of each nuclear receptor gene. This allowed us to categorize nuclear receptors into two functional classes –25 nuclear receptors which we hypothesize to be targets of long-range regulation (cluster 1 in Table 1), and remaining 23 nuclear receptors which are not targets (cluster 2). We discuss our key findings below.

A number of developmental genes are present in cluster 1, including some that are known targets of long-range gene regulation. On the other hand, cluster 2 contain several genes which are tissue-specific and consequently do not utilize long-range regulation. Further, genes present in cluster 1 have longer and often multiple CpG islands, a known characteristic of target genes under the GRB model.

We have also identified cases of multiple nuclear receptors present in the same GRB locus (Figure S3). It is not unusual to have GRBs with multiple targets – HOX, IRX and DLX loci are known examples - and at least some GRB targets that occur in separate loci in vertebrates are found next to each other in e.g. *Drosophila* genome [28]. However, this makes it hard to predict which of the genes present in the same locus are being regulated. To address this, we used other promoter-related features, e.g. presence of bivalent domain, which are known to be present in genes having long-range regulation (Figure 5). Our analysis provides strong indication as to which genes are the targets of long-range regulation and therefore, can be used when investigating other GRBs with multiple targets.

To further validate our results, we have investigated the impact of different individual histone modifications. We found that genes present in cluster 1 have significantly higher enrichment of enhancer mark (H3K4me1) around their gene loci compared to genes in cluster 2 (Figure 4), indicating multiple enhancers including those overlapping HCNEs. Subsequent analysis of repressive marks (H3K27me3) reveals that several genes in cluster 1 have bivalent domain in their promoter regions (Figure 5). This provides further indication that these genes require spatio-temporal control of their transcription facilitated by gain/loss of active and repressive promoter marks. Further experimental study using time-series data can elucidate this phenomenon.

We also studied combinatorial patterns of histone modifications, which have been shown to capture functional dynamics associating with specific biological functions of the genome [33]. We note that our original categorization is recovered (except for two genes, see Results for details) using this approach, lending crucial evidence that long-range regulation (captured by our method) is key to the functional roles of more than half of the nuclear receptors.

**Figure 7:**
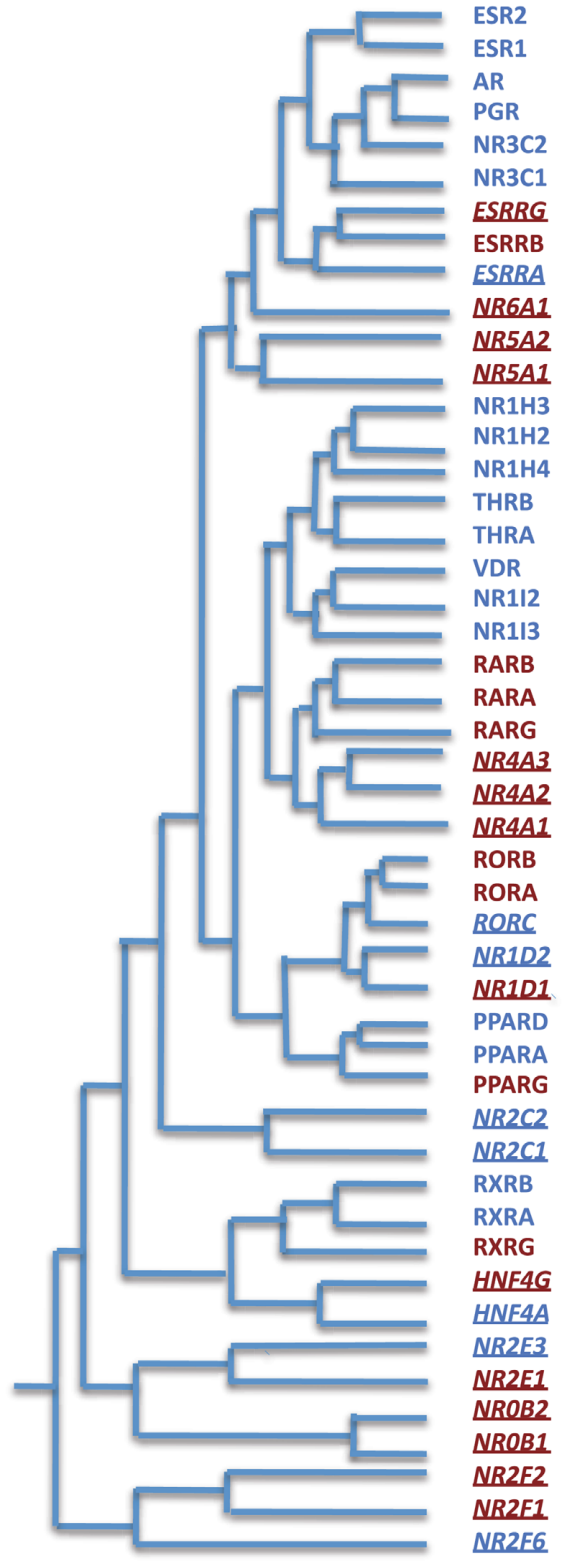
Classification comparison of nuclear receptors gene family with respect to sequence homology and transcriptional mechanism and function based. The GRB target genes (cluster 1 in Table 1) are shown in red, while non-targets are in blue. Nuclear hormone receptors are presented in normal bold text while orphan receptors are underlined and in italics. There are in total 23 nuclear receptor GRB target genes and 25 nuclear receptor non-GRB target nuclear receptor genes. It is clear from the figure that both GRB target and non-target nuclear receptors are dispersed among seven families classified on the basis of sequence homology.

Figure 7 presents our final classification of nuclear receptors into possible targets of long-range regulation (shown in red) and non-targets (shown in blue) taking into account presence of multiple targets in the same GRB loci. We show sequence-based similarity, highlighting the fact that new paralogs in evolution often acquire a different mode of regulation. Following further with above classification, investigation of evolutionary mechanism whereby the paralogs acquired different regulation is the logical next step. We expect nuclear receptors implicated to be targets of long-range regulation have likely evolved by whole genome duplication events, and therefore, retained their regulatory inputs over a wide region. In contrast, other nuclear receptors possibly evolved through more localized (tandem) duplications.

## Materials and Methods

### HCNE based Analysis and CpG Islands Detection

We have used the following genome assemblies for this study: human (hg19), mouse (mm10), chicken (galGal4), fugu (fr3) and zebrafish (Zv9). All the gene coordinates were obtained from Ensembl ([42]; http://www.ensembl.org; version 72) using Biomart (http://www.biomart.org). The associated scripts are available at http://www.bitbucket.org/yogita_sharma/nr_classification/.

The genomic coordinates of HCNEs were obtained from the Ancora genome browser ([43]; http://ancora.genereg.net). The selected conservation threshold and length cut offs for each species are specified in Table S1. The CpG island locations were downloaded from the UCSC Genome Table Browser ([44]; http://genome-euro.ucsc.edu/cgi-bin/hgTables?hgsid=194624867 *).* For each pair-wise comparison between human and one of the other genomes, we computed the HCNEs ±2 Mb region of each nuclear receptor gene loci. This is to capture *cis*-regulatory elements, which may occur far from the gene location.

The extension of genomic co-ordinates around each gene loci for HCNE detection might create biasness towards the longer genes. To avoid this we normalized the obtained HCNE counts with respect to the gene length. The log2 values of the HCNE counts were used to compute the dissimilarity matrix for all the genes across different five genomes (Euclidean distance measure). Finally we performed the hierarchical clustering, using complete linkage, to analyze the HCNEs across the gene set. This method is more robust to outliers compared to classification based on a single threshold such as mean etc.

The CpG island locations were downloaded from the UCSC Genome Table Browser [44]. For this analysis, we used three gene sets; nuclear receptors, transcription factors and CpG genes. The ±1 kb flanking region around all the genes were scanned to count the total number of CpG base pairs. Along with the calculation of CpG island number we also calculated the total CpG island lengths for the gene sets. The cumulative distributions of the CpG island length were plotted for all the genes.

We also compared the HCNE counts between nuclear receptors and other random selected transcription factors. We randomly selected 48 genes out of around 900 (Table S4, Sheet 2) using GNU R function sample() with default seed and burn-in of 500. We obtain transcription factor gene coordinates from the Ensembl database (version 72). To be able to compare between the different gene sets we pooled the randomly selected set of genes with the nuclear receptor gene family and repeated previous experiment. The HCNEs were calculated and plotted in the same way as in the previous experiment.

### RNA-seq Data

The RPKM files for expression-based analysis (RNA-seq) was downloaded from ENCODE ([31]; http://genome-euro.ucsc.edu/ENCODE/downloads.html) for five cell lines (Gm12878, H1hesc, Huvec, HepG2, k562) for hg18 genome assembly.

### ChIP-seq Data

The tag aligned files downloaded for five cell lines (Gm12878, H1hesc, Huvec, HepG2, k562) from hg18 genome assembly of ENCODE [31] project were used for the peak calling. We extracted the significant enriched regions between chip versus control using CCAT package [45]. Standardized settings (fragmentSize = 200, isStrandSensitiveMode = 0, slidingWinSize = 500, movingStep = 50, outputNum = 100000, minCount = 4, minScore = 3.0, bootstrapPass = 50, randSeed = 123456) were implemented for the analysis. Finally top 10,000 peaks (with p-value <0.001) were used for further downstream analysis. After preprocessing the data set we extracted coverage (vector representing read per million values for each bin) across different genomic ranges of interest. To be able to compare across different cell lines we normalized the coverage across the dataset by dividing obtained coverage w.r.t. library size. Table S5 presents the genomic ranges used for analysis of different histone marks [46], [47], [48].

### Statistical Significance Test for Enhancer Data

To check the significance of the difference obtained in enrichment of H3k4me1 mark across both clusters, we performed statistical testing against background set as follows: We extracted a set of 2054 genes (chromosome X in hg18 genome assembly) from Ensembl database using the R library (biomaRt). Subsequently, we classified this gene set based on presence of CpG island within ±1 kb region of transcription start site of each gene; obtaining a candidate set of 402 genes with CpG islands, and the remaining set of 1652 genes without CpG islands.

We constructed the background set consisting of 2054 genes obtained as described above as well as the set of 48 nuclear receptor genes, resulting in a total size of 2102 genes. We drew 1000 bootstrap samples from this set, and for each sample, we counted the number of reads overlapping regions of different width (±10 kb, ±1 Mb and ±2 Mb) around the transcription start site for each gene. This was used to construct background distribution of the number of reads for each of the different region widths (respectively ±10 kb, ±1 Mb and ±2 Mb).

We have also extracted a set of 1455 genes on chromosome 5 and classified the gene set in to CpG (650) and non-CpG genes (805) on the basis of presence/absence of CpG island. We performed the statistical analysis in the similar way as mentioned above.

### Chromatin State Map Analysis

Chromatin state map is a hidden Markov model-based mapping of different chromatin states across the different cell lines [33]. The data was downloaded from UCSC genome browser [44]. Since we were interested to see the difference in regulatory content of developmental related and non-related nuclear receptor genes, we only considered the embryonic stem cell line (H1hesc) data for this analysis. We calculated the total number of state counts for each gene in all the states across selected genomic ranges in H1hesc. We used different random genomic ranges (±10 kb and ±100 kb around TSS) to study the enrichment of chromatin states. To see the combinatorial patterns of histone modifications around all genes we prepared a heatmap using log2 ratio of the number of state counts for each gene using the default parameters (Hierarchical clustering with full/complete linkage using Euclidean distance measure).

## Supporting Information

Figure S1 **Cumulative distribution plots of total CpG island length across three data sets.** The GRB targets nuclear receptors have longer CpG islands than randomly selected CpG and transcription factor genes. The GRB target NR, random selected transcription factors and CpG genes are presented in green, red and black, respectively.
(EPS)

Figure S2 **Clustering of genes based on HCNE counts in augmented set of nuclear receptors and randomly selected transcription factors.** The nuclear receptors in cluster 1 (Table 1) are present in the same cluster here as well.
(EPS)

Figure S3 **Cases of multiple targets present in same GRB locus.** A) Block of three genes (*THRB, RARB* and *NR1D2*) in human on chromosome 3 and their 1-to-1 orthologs in mouse in chromosome 14. B) Block of three genes (*THRA, RARA* and *NR1D1*) in human on chromosome 17 and their 1-to-1 orthologs in mouse in chromosome 11. C) Block of two genes in human (*NR6A1, NR5A1*).
(EPS)

Figure S4 **H3K4me3 average coverage plot for nuclear receptor genes in cluster 1 (putative targets of long-range regulation).** The average H3K4me3 coverage plots around ±10 kb TSS across different cell lines when genes are expressed (left) and not expressed (right). The x-axis shows position around ±10 kb TSS and y-axis represent average coverage. It shows when genes are expressed they have peak of active promoter around their TSS. Different colors represent different cell lines.
(EPS)

Figure S5 **H3K4me3 average coverage plots for nuclear receptor genes in cluster 2 (non-targets based on GRB model).** The average H3K4me3 coverage plots around ±10 kb TSS across different cell lines when non-GRB target genes are expressed (left) and not expressed (right). The x-axis shows position around ±10 kb TSS and y-axis represent average coverage. Expressed genes have active promoter signal around their TSS. Different colors represent respective cell lines.
(EPS)

Figure S6 **UCSC genome browser view of promoter region of selected five cases from Cluster 1 genes.** The promoter region of five (*NR4A1, NR5A2, NR1D1, RORB* and *ESRRG*) genes around ±5 KB TSS. The direction of arrow represents transcription direction. The first peak corresponds to active transcription (H3K4me3) followed by the peak of repression mark (H3K27me3) in the track below. CpG islands are shown in green.
(EPS)

Figure S7 **Average coverage plots of repression mark (H3k27me3) around different clusters.** The x-axis shows position around ±10 kb TSS and y-axis coverage. Cluster 1 (red color) has higher coverage of repression mark in comparison to cluster 2 (green color). The blue line represents TSS.
(EPS)

Figure S8 **Statistical significance test for H3K4me1 around different genomic distributions on chromosome 5.** A) H3K4me1 distribution in different clusters across ±10 kb TSS against the random background distribution. B) H3K4me1 distribution in different clusters across ±1 Mb TSS with respect to random background distribution. C) H3k4me1 distribution in different clusters across ±2 Mb TSS with respect to random background distribution. This figure shows that cluster 1 (shown by red bar) has significantly higher distribution of H3K4me1 in comparison to random selected background region (marked by black bars), CpG and non-CpG region (shown by blue and green bar respectively) and cluster 2 genes (shown by pink bar).
(EPS)

Table S1 **The percentage of conservation and length cut offs for HCNE counts.**
(DOC)

Table S2 **The list of genes in HCNE based clustering of augmented set consisting of 48 nuclear receptors and 48 randomly selected transcription factors.** Known targets of long-range gene regulation are marked with asterisk (*).
(DOC)

Table S3 **The RPKM values of each nuclear receptor gene across 5 cell lines.**
(XLS)

Table S4 **List of HMM states associated with specific functional elements of the genome.**
(XLS)

Table S5 **The genomic ranges for different histone modifications.**
(DOC)
