# Supplementary material for: Computational Characterization of Modes of Transcriptional Regulation of Nuclear Receptor Genes
Source: PLoS One. 2014 Feb 13;9(2):e88880. doi: 10.1371/journal.pone.0088880 (PMC3923872; doi:10.1371/journal.pone.0088880)
Supplement: Table S1 — The percentage of conservation and length cut offs for HCNE counts. (DOC) [file pone.0088880.s009.doc]

**Supplementary Table 1. The percentage of conservation and length cut offs for HCNE densities.**

| HCNEs | Conservation Threshold | Length cut off |
| --- | --- | --- |
| Human – Mouse | 98% | 50 |
| Human – Chicken | 98% | 50 |
| Human – Frog | 90% | 50 |
| Human – Fugu | 70% | 50 |
| Human – Zebrafish | 70% | 50 |
