# Supplementary material for: Computational Characterization of Modes of Transcriptional Regulation of Nuclear Receptor Genes
Source: PLoS One. 2014 Feb 13;9(2):e88880. doi: 10.1371/journal.pone.0088880 (PMC3923872; doi:10.1371/journal.pone.0088880)
Supplement: Table S2 — The list of genes in HCNE based clustering of augmented set consisting of 48 nuclear receptors and 48 randomly selected transcription factors. Known targets of long-range gene regulation are marked with asterisk (*). (DOC) [file pone.0088880.s010.doc]

**Supplementary Table 2**. **The list of genes in HCNE based clustering of augmented set consisting of 48 nuclear receptors and 48 randomly selected transcription factors genes.**

| **Cluster A** | | | | **Cluster B** | | | |
| --- | --- | --- | --- | --- | --- | --- | --- |
| **Nuclear receptors** | | **Other TFs** | | **Nuclear receptors** | | **Other TFs** | |
| NR1D1 | PPARG* | PAX2* |  | NR1H3 |  | ZNF695 | RUNX3 |
| RARA | NR2F2* | SOX2* |  | AR |  | MGA | RFX7 |
| THRA |  | MEIS2* |  | NR2C1 |  | ZBTB40 | GABPA |
| NR4A3 |  | OSR2 |  | RORC |  | ZNF519 | TEAD3 |
| NR6A1 |  | CDX2 |  | NR2E3 |  | ZNF131 | ZSCAN20 |
| NR1D2 |  | PDX1 |  | NR1I2 |  | ELF1 | ZNF275 |
| RARB |  |  |  | NR1H4 |  | MIER1 | CREB3L1 |
| THRB |  |  |  | ESR1 |  | HIVEP1 | GRHL3 |
| RARG |  |  |  | ESR2 |  | ZNF24 | ZNF239 |
| HNF4G |  |  |  | NR2C2 |  | ZNF396 | ZNF663 |
| NR0B1 |  |  |  | NR1H2 |  | ZNF271 | PPP1R3E |
| NR2E1 |  |  |  | PGR |  | ZNF397 | TCF19 |
| NR5A1 |  |  |  | RXRA |  | ZNF81 | ZNF409 |
| RORA |  |  |  | NR3C1 |  | ZNF589 | GATA6 |
| RORB |  |  |  | NR3C2 |  | SMAD9 | E2F2 |
| NR0B2 |  |  |  | PPARD |  | SOX13 | ZNF74 |
| NR4A2 |  |  |  | VDR |  | ZNF204 | ZNF683 |
| ESRRG |  |  |  | NR1I3 |  | ZNF187 | ZNF436 |
| NR5A2 |  |  |  | RXRB |  | PRDM2 |  |
| NR2F1 |  |  |  | NR2F6 |  | GMEB1 |  |
| ESRRB |  |  |  | PPARA |  | BACH1 |  |
| NR4A1 |  |  |  | HNF4A |  | ZBTB17 |  |
| RXRG |  |  |  | ESRRA |  | LZTR1 |  |
