# Supplementary material for: Computational Characterization of Modes of Transcriptional Regulation of Nuclear Receptor Genes
Source: PLoS One. 2014 Feb 13;9(2):e88880. doi: 10.1371/journal.pone.0088880 (PMC3923872; doi:10.1371/journal.pone.0088880)
Supplement: Table S5 — The genomic ranges for different histone modifications. (DOC) [file pone.0088880.s013.doc]

**Supplementary Table 5 . The genomic ranges for different histone modifications.**

| Histone Modification | Genomic Range |
| --- | --- |
| H3k4me2, H3k4me3, H3k27me3 | ±10kb TSS |
| H3k36me3 | ±20kb mid of the gene |
| H3k4me1 | ±10kb, ±50kb, ±1Mb, ±2Mb TSS |
